# Circular RNA circSIPA1L1 Contributes to Osteosarcoma Progression Through the miR-411-5p/RAB9A Signaling Pathway

**DOI:** 10.3389/fcell.2021.642605

**Published:** 2021-04-22

**Authors:** Yining Xu, Teng Yao, Haonan Ni, Rujie Zheng, Kangmao Huang, Yizhen Huang, Jun Gao, Di Qiao, Shuying Shen, Jianjun Ma

**Affiliations:** ^1^School of Medicine, Shaoxing University, Shaoxing, China; ^2^Key Laboratory of Musculoskeletal System Degeneration and Regeneration Translational Research, Zhejiang University School of Medicine, Hangzhou, China; ^3^Kunming Medical University, Kunming, China; ^4^The First Affiliated Hospital of Wenzhou Medical University, Wenzhou, China

**Keywords:** circular RNA, microRNA, osteosarcoma, circSIPA1L1, miR-411-5p, RAB9A

## Abstract

Recently, various studies have identified circular RNAs (circRNAs) to play a significant role in tumorigenesis, thereby showing potential as novel tumor biomarkers. circSIPA1L1 is a newly discoveredcircular RNA, which is formed by back-splicing of SIPA1L1 and is found increased in osteosarcoma (OS). Nevertheless, the specific functions of circSIPA1L1 in OS remain unknown. In the present study, circSIPA1L1 was obtained from a previously reported circRNA microarray in the GEO database (GSE96964). Quantitative real-time polymerase chain reaction (qRT-PCR) was employed to assess the mRNA level of circSIPA1L1 in OS cell lines and tissue samples. Bioinformatics analysis, luciferase reporter assays, real-time PCR, RNA pull-down assays and RNA immunoprecipitation (RIP) were employed to verify the binding of circSIPA1L1 with miR-411-5p. Xenograft tumor models were established to identify the role of circSIPA1L1 *in vivo*. A series of *in vitro* experiments, such as western blotting, colony formation, transwell assays and anoikis assay were employed to confirm the relationship across circSIPA1L1, miR-411-5p, and RAB9A. Our study confirmed circSIPA1L1 to be upregulated in both human OS samples and OS cell lines. Mechanistically, circSIPA1L1 could serve as a miR-411-5p molecular sponge to increase RAB9A expression, which was confirmed to be a tumor promoter mediating carcinogenesis. Silencing of circSIPA1L1 attenuated the vitality, invasion, migration and proliferation of OS cell lines both *in vivo* and *in vitro*. miR-411-5p inhibition or RAB9A overexpression reversed the anti-tumor effects caused by circSIPA1L1 knockdown. Briefly, circSIPA1L1 could function as a driver gene in OS and initiate OS tumorigenesis through the miR-411-5p/RAB9A signaling pathway, which might become a potential therapeutic biomarker for OS treatment.

## Introduction

Osteosarcoma (OS), a neoplasm with high aggressiveness and formidable distant metastatic characteristics, is the third most prevalent and lethal primary bone malignancy across children, youth, and adolescents (Ritter and Bielack, 2010). At present, the major therapeutic strategy proposed for patients suffered from OS is surgical resection and polychemotherapy in combination (Kansara et al., 2014). Nevertheless, owing to the rapid proliferation, high metastatic potential, and multi-drug resistance of OS, the prognosis of patients with metastatic OS remains poor (Harrison et al., 2018). According to previous reports, while the long-term overall survival of patients suffered from OS with no metastasis is greater than 60%, the 5-year survival rate of patients in an advanced disease stage could be as low as 20%, owing to distant metastasis and recurrence (Gianferante et al., 2017; Kumar et al., 2018). Therefore, investigation of the underlying molecular mechanisms and identification of highly sensitive and clinically relevant markers of OS are urgently required for early diagnosis and development of more effective therapeutic measures.

As an attractive class of non-coding endogenous RNA transcript, circular RNA (circRNA) is being extensively investigated due to its unique circular structure formed by non-canonical splicing of linear pre-mRNAs (Du et al., 2017; Kristensen et al., 2019). circRNA is characterized by lacking 5′ caps and 3′ polyadenylated tails, and is equipped with covalently closed continuous loop structure, which make it highly stable and resistant to degradation by endonucleases (Patop et al., 2019). In recent years, studies have shown that circRNAs exert significant influence on multiple human diseases by interacting with RNA-binding proteins, sponging microRNA (miRNA), and regulating transcription and protein translation (Hsiao et al., 2017; Li H.M. et al., 2019; Mahmoudi et al., 2019; Yu et al., 2019). With advancement in high-throughput sequencing and bioinformatics approaches, a growing number of circRNAs have been demonstrated to be highly expressed in multiple human cancers, and to play crucial roles in a wide variety of biological processes in cancer (Han et al., 2018; Cheng et al., 2019). Nevertheless, the specific function and mechanisms of circSIPA1L1 in OS have not been adequately elucidated.

miRNAs form another class of non-coding RNAs that have been broadly investigated (Lu and Rothenberg, 2018). Substantial studies have identified the vital role of miRNAs in regulating various kinds of biological functions, including gene expression, cell proliferation, cell cycle, apoptosis, and differentiation (Yu et al., 2018; Yuan and Weidhaas, 2019). While miR-411-5p has been discovered to be firmly correlated with prognosis in hepatocellular carcinoma (Liu et al., 2018), few reports have identified its role in OS.

Originated from the Rab GTPase family, RAB9A is essential to the recycling of mannitose 6-phosphate receptors from late endosomes to trans-Golgi network (Sun et al., 2020). RAB9A is also involved in lysosomal biogenesis and late endosomal morphology (Mahanty et al., 2016). RAB9A is known to be highly detected in breast cancer samples and is tightly linked to the biological progression of breast cancer (Liu et al., 2019). But whether it could be targeted by circSIPA1L1 and miR-411-5p in OS needs to be explored further.

In this study, we confirmed circSIPA1L1 from exons 5 and 7 of the *SIPA1L1* gene and *RAB9A* as oncogenes in OS. The expression of circSIPA1L1 was upregulated in OS, although the mRNA level of *SIPA1L1* remained unchanged. In conclusion, circSIPA1L1 remarkably increased the proliferation and metastasis of OS through the miR-411-5p/RAB9A signaling pathway.

## Materials and Methods

### Ethical Approval

All experiments involving animals were conducted following the guidelines of the Guide for the Care and Use of Laboratory Animals published by the National Institutes of Health and ratified by the Ethics Committee of the Sir Run Run Shaw Hospital.

### Clinical Samples

Ten pairs of fresh frozen chondroma tissue samples and OS tissue samples from 20 untreated patients before surgery (10 for each group), were acquired from the Sir Run Run Shaw Hospital between March 2019 and March 2020. All patients offered written informed consent approving the samples for research. Our project was authorized by the Ethics Committee of the Sir Run Run Shaw Hospital. The ethic number was 20210218-30. All samples were stored at liquid nitrogen until RNA extraction.

### Cell Culture

Normal osteoblast cell hFOB1.19 and human OS cell lines, including 143B, SJSA-1, HOS, MG-63, U2OS were purchased from ATCC. All the cell lines were grown in Dulbecco’s modified Eagle medium (DMEM) supplemented with 10% fetal bovine serum (FBS). All the cells were identified to be mycoplasma-free and were kept at 37°C with 5% CO_2_.

### Cell Transfection

Short hairpin RNA targeting (siRNAs) targeting circSIPA1L1, miR-411-5p mimics and miR-411-5p inhibitors, RAB9A overexpression plasmid and their negative controls were obtained from RiboBio (Guangzhou, China). The plasmids and other vectors were transfected into OS cells using Lipofectamine 3000 (Thermo Fisher Scientific, IL, United Staes). After transfected with negative controls or plasmids for 48–72 h, the OS cells were harvested and applied to qRT-PCR to evaluate the efficiency of transfection. The sequences of siRNAs and ShRNAs used in the cell transfection were shown in the Supplementary Table 2. To generate different kinds of 143B stable cells, a variety of lentiviruses were employed according to different requirements. Human lentivirus-sh-circSPIA1L1, Human lentivirus-negative control and Human lentivirus-miR411-5p sponge were obtained from Hanbio. In brief, the culture medium was supplemented with the lentiviruses.48 hours after infection, the 143B cells were sorted with 2 μg/ml puromycin in culture medium for 72 hours, and the surviving 143B cells were collected for further experiments.

### RNA Isolation, RNase R Digestion, and qRT-PCR

Total RNAs were extracted using Trizol reagent (Invitrogen) according to the manufacture protocols. For RNase R treament, 2 mg of total RNA deriving from OS cell lines was treated with or without RNase R (Epicentre Technologies) for 15 min at 37°C. To synthesize the complementary DNA (cDNA), a PrimerScript RT reagent kit (TaKaRa) was employed. qRT-PCR were subsequently conducted using SYBR Premix ExTaq II (TaKaRa). GAPDH was served as a normalization for mRNA and circRNA analyses. U6 was employed as an internal standard control for miRNA analyses. The primer sequences used in research were exhibited in the Supplementary Table 2.

### Xenograft Tumorigenesis

The design of this experiment has been previously reported by Liu et al. (2018). In brief, 4-week-old nude mice (male) were prepared for the xenograft tumor models. Subsequently, the animals were divided into three groups at random. 4 × 10^6^ 143B stable cells, which were transfected with sh-NC (negative control), or sh-circSIPA1L1, or sh-circSIPA1L1 and miR-411-5p sponge were suspended in phosphate-buffered saline (PBS) and were injected subcutaneously. Tumor volumes were calculated with formula listed below: volume = (width × length)^2^/2. Thirty days after treatment, the nude mice were sacrificed, and the weight and volume of their tumors were measured.

### Fluorescence *in situ* Hybridization

Cy3-labeled circSIPA1L1 probes were purchased from RiboBio. A Fluorescent *in situ* Hybridization Kit was employed to detect the signals of the probes following the kit instructions. The FISH sequence for circSIPA1L1 was as follows: 5′CY3-AGGGAGAAAGCATGGGATTATG 3′. The images were captured using Nikon A1Si Laser Scanning Confocal Microscope (Japan).

### RNA Immunoprecipitation

RNA Immunoprecipitation (RIP) assays were employed utilizing the Magna RIP Kit (Millipore). Sh-NC or sh-circSIPA1L1 was stably transfected into 143B cells. Subsequently, 143B cells were lysed using RIP lysis buffer supplemented with RNase inhibitors and protease inhibitors. Then IgG antibody or anti-Ago2 coated beads (Millipore) were used to incubate with lysates with rotation overnight at 4°C. The lysates were treated with proteinase K buffer, followed by RNA extraction. Purified RNA was acquired and reversely transcribed. Finally, qRT-PCR was performed to detect the abundance of circSIPA1L1.

### Pull-Down Assay

In brief, 1 × 10^7^ 143B and HOS cells were treated with lysis buffer. C-1 magnetic beads were incubated with circSIPA1L1 probe for 2 h at 25°C to acquire probe-coated beads. After that, oligo probe or circSIPA1L1 probe was incubated with the cell lysates overnight at 4°C, respectively. The RNA compounds binding to the magnetic beads were washed four times with washing buffer. After purification, RT-PCR and qRT-PCR were employed to detect the expression levels of candidate miRNAs.

### Luciferase Reporter Assay

HEK-293 cells were seeded into 96-well plate and cultured overnight. Subsequently, the cells were co-transfected with luciferase reporter plasmid mixtures using LipofectamineTM2000 reagent. After 24 h, the luciferase activity was detected with a dual-luciferase reporter assay system (Promega).

### Western Blotting Analysis and Antibodies

RIPA lysis buffer with 1% protease inhibitor (Fudebio) was utilized to lyse stable transfected OS cells. Subsequently, the concentrations of protein were evaluated using a BCA Protein Assay Kit (Beyotime). The protein samples were separated using 10% SDS-PAGE by electrophoresis, and then were transferred onto a PVDF membrane (Millipore) and blocked with 5% non-fat milk powder in TBST for 2 h. Specific primary antibodies were incubated with the membranes at 4°C overnight. After washing with TBST, the membranes were treated with a secondary antibody (Fudebio) for 1 h. Finally, an ECL substrate kit (Thermo Fisher Scientific, IL, United States) was employed to visualize the immunoblots results. The primary antibodies employed in this study were as follows: β-actin (Abcam); RAB9A (Cell Signaling Technology); E-cadherin and N-cadherin (Proteintech).

### Wound-Healing Assay

Transfected OS cells were cultured in 6-well cell culture plates and cultured overnight until 90% confluence, and the confluent cell layer were scrape a straight scratch with 200 μl pipette tips. Representative images of cell migration from specific scratched positions were captured by microscopy at 0 and 24 h after injury.

### Colony Formation Assay

Briefly, 143B and HOS cells (5 × 10^2^ per well) were seeded into a 12-well cell culture dishes and cultured for 10 days. After washing with PBS twice, the colonies were fixed using 4% paraformaldehyde for 15 min and stained with 0.1% crystal violet for another 15 min at 37°C. The colonies were counted and photographed using a microscope.

### Migration-Related Assays

Regarding to transwell migration assay, the transfected 143B and HOS cells were cultured in 200 μL of serum-free medium and added into the upper chambers (8 μm, Corning, NY, United States). While the lower chambers were supplemented with 500 μl fresh culture medium containing 10% FBS. Invasion assays were performed in the same procedure except that the membrane was covered with Matrigel. After incubating for 24 h, the migrated and invaded cells were fixed and subjected to 0.1% crystal violet solution staining. The images were captured using a microscope depending on at least five random fields.

### Apoptosis Analysis

Apoptosis analysis was conducted using an AnnexinV-FITC/PI double staining kit (BD Biosciences). Briefly, the transfected OS cells were harvested and subjected to resuspension in 1 × Binding Buffer and then treated with Annexin V-FITC/PI according to the kit instructions. After incubating for 15 min, a flow cytometer (BD Biosciences) was employed to detect the Apoptosis of the cells.

### Statistical Analyses

Statistical analyses were carried out using SPSS 20 software. Two groups experimental data were evaluated with unpaired Student’s *t*-test. Multiple groups were compared with one-way analysis of variance (ANOVA). *P*-values less than 0.05 indicated that the data were statistically significant.

## Results

### circSIPA1L1 Was Expressed Relatively Abundantly in OS Cell Lines and Tissues and Mainly Localized in the Cytoplasm

**FIGURE 1:**
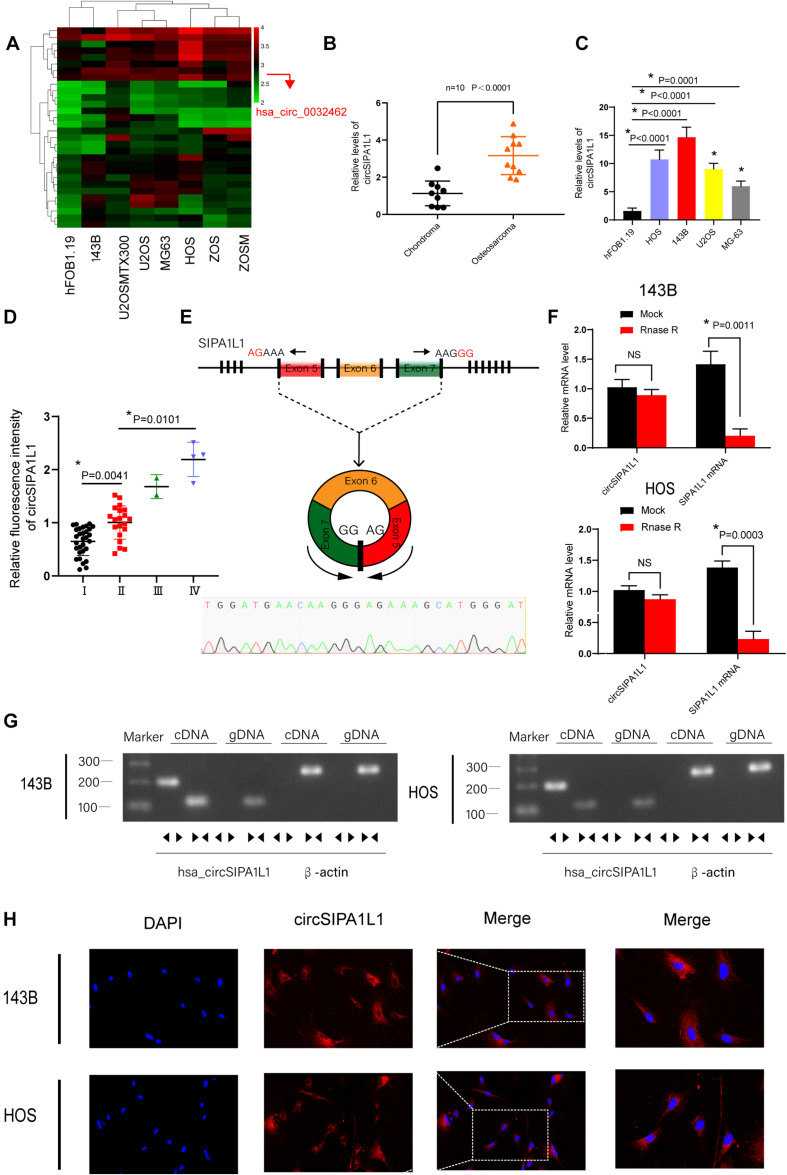
Expression and validation of circSIPA1L1 in osteosarcoma (OS) cells and tissue. **(A)** Heatmap for differentially expressed circRNAs based on hFOB1.19 and OS cell lines in GSE96964. **(B)** circSIPA1L1 expression in 10 OS and chondroma tissue samples as detected using qRT-PCR (*n* = 10). **(C)** The expression of circSIPA1L1 was upregulated in a series of OS cell lines (HOS, 143B, U2OS, and MG-63) compared with hFOB1.19 cells. **(D)** The mRNA levels of circSIPA1L1 in OS at different clinical stage was detected by FISH assays on an OS tissue sample chip (56 samples in all). *P < 0.05. **(E)** Schematic illustration exhibited the circularization of exons 5–7 in SIPA1L1 forming circSIPA1L1 by back splicing. The existence of circSIPA1L1 was proved by Sanger sequencing. **(F)** The expression of circSIPA1L1 and SIPA1L1 mRNA in 143B and HOS cells was evaluated by RT-PCR, with or without RNase R treatment. **(G)** The existence of circSIPA1L1 was verified in 143B and HOS cells. circSIPA1L1 was amplified using divergent primers in cDNA rather than genomic DNA. GAPDH was employed as a negative control. The same or the opposite directions of the arrowhead represented divergent primers or convergent primers. **(H)** FISH assay showed circSIPA1L1 to mainly localize in the cytoplasm. Data from three independent experiments are presented as mean ± standard deviation (SD). *P < 0.05.

First, we downloaded a microarray expression dataset (GSE96964) from the GEO database (Table 1), comparing circRNAs in hFOB1.19 cells with those in OS cells to select the differentially expressed circRNAs. We discovered circSIPA1L1 (also called circ_0032462) to be highly expressed in a series of OS cells than in normal osteoblast cell line (Figure 1A). To determine the specific function of circSIPA1L1 in OS progression, 10 pairs of chondroma and OS tissue samples were collected to measure the expression level of circSIPA1L1 using qRT-PCR. Results indicated the abundance of circSIPA1L1 to be remarkably enhanced in OS tissue samples compared with chondroma tissue samples (Figure 1B). We next validated using qRT-PCR that the abundance of circSIPA1L1 was notably increased in OS cell lines (U20S, 143B, HOS, and MG-63) in comparison with hFOB1.19 cells. Among the various OS cell lines, 143B and HOS cells showed significant increase in circSIPA1L1 expression (Figure 1C). Figure 1D demonstrated that the expression of circSIPA1L1 was positively related with OS clinical stages. Divergent primers of circSIPA1L1 were designed, and head-to-tail splicing was identified in the RT-PCR-amplified product of circSIPA1L1 (Figure 1E). Also, the result was identified by Sanger sequencing. Stability is a significant feature of circRNAs that differentiates them from mRNAs. To verify the stability of circSIPA1L1, RNase R was used in the assay. circSIPA1L1 exhibited high tolerance to RNase R treatment while the levels of linear form of SIPA1L1 were significantly decreased upon exposure to RNase R (Figure 1F). To eliminate the possibility of trans-splicing or genomic rearrangements generating head-to-tail splicing, gDNA and cDNA were acquired from 143B and HOS cells, followed by DNA electrophoresis. While circSIPA1L1 could only be detected from cDNA, SIPA1L1 could be detected from both gDNA and cDNA (Figure 1G). FISH assays indicated circSIPA1L1 to be predominantly localized in the cytoplasm (Figure 1H).

**TABLE 1:** The circ ID in red is the circRNA (has_circ_0032462) we employed in our study.

circ_ID	Adj. *P*-value	*P*-value	*t*	*B*	logFC
ASCRP001659	5.789508	11.59767	–1.53964	–4.51339	2.57335
ASCRP001660	0.9999	0.166445	–1.50411	–4.55085	2.25283
ASCRP003119	0.9999	0.365631	–0.95214	–5.06052	2.17437
ASCRP001480	0.9999	0.304625	–1.08788	–4.94992	2.00213
ASCRP002384	0.9999	0.423205	–0.83855	–5.14413	1.76108
ASCRP000064	0.9999	0.107415	–1.78583	–4.24315	1.70778
ASCRP001750	0.9999	0.018981	–2.84693	–2.98806	1.69917
ASCRP002932	0.9999	0.480806	–0.73508	–5.21256	1.60013
ASCRP004706	0.9999	0.242608	–1.24967	–4.8045	1.56409
ASCRP001505	0.9999	0.009889	–3.24783	–2.52448	1.54482
ASCRP002211	0.9999	0.060967	–2.13727	–3.83474	1.53514
ASCRP001589	0.9999	0.177516	–1.46158	–4.59509	1.51367
ASCRP000361	0.9999	0.243212	–1.24794	–4.80612	1.5101
ASCRP004540	0.9999	0.398642	–0.88558	–5.11055	1.50155
ASCRP002386	0.9999	0.483885	–0.72979	–5.21585	1.49749
ASCRP002787	0.9999	0.142979	–1.60324	–4.44529	1.49686
ASCRP003022	0.9999	0.127856	–1.67517	–4.36675	1.46795
ASCRP000629	0.9999	0.080166	–1.96867	–4.03307	1.45847
ASCRP002683	0.9999	0.243093	–1.24828	–4.8058	1.4515
ASCRP001839	0.9999	0.163964	–1.51398	–4.54049	1.44715
ASCRP004073	0.9999	0.231143	–1.28319	–4.7727	1.40522
ASCRP000631	0.9999	0.03454	–2.4832	–3.42128	1.37071
ASCRP002802	0.9999	0.2673	–1.18167	–4.86729	1.34898
ASCRP001736	0.9999	0.395819	–0.89112	–5.1065	1.32812
ASCRP005305	0.9999	0.030355	–2.56156	–3.32742	1.28616
ASCRP004578	0.9999	0.136322	–1.63401	–4.41187	1.28534
ASCRP004091	0.9999	0.103051	–1.81196	–4.21353	1.2764
ASCRP001277	0.9999	0.171346	–1.48499	–4.57083	1.27041
ASCRP002643	0.9999	0.154531	–1.55277	–4.49945	1.26456
ASCRP000307	0.9999	0.286322	–1.13266	–4.91106	1.24367

### circSIPA1L1 Knockdown Inhibited the Invasion, Migration, Proliferation, and Survival Ability of OS Cells *in vitro*

**FIGURE 2:**
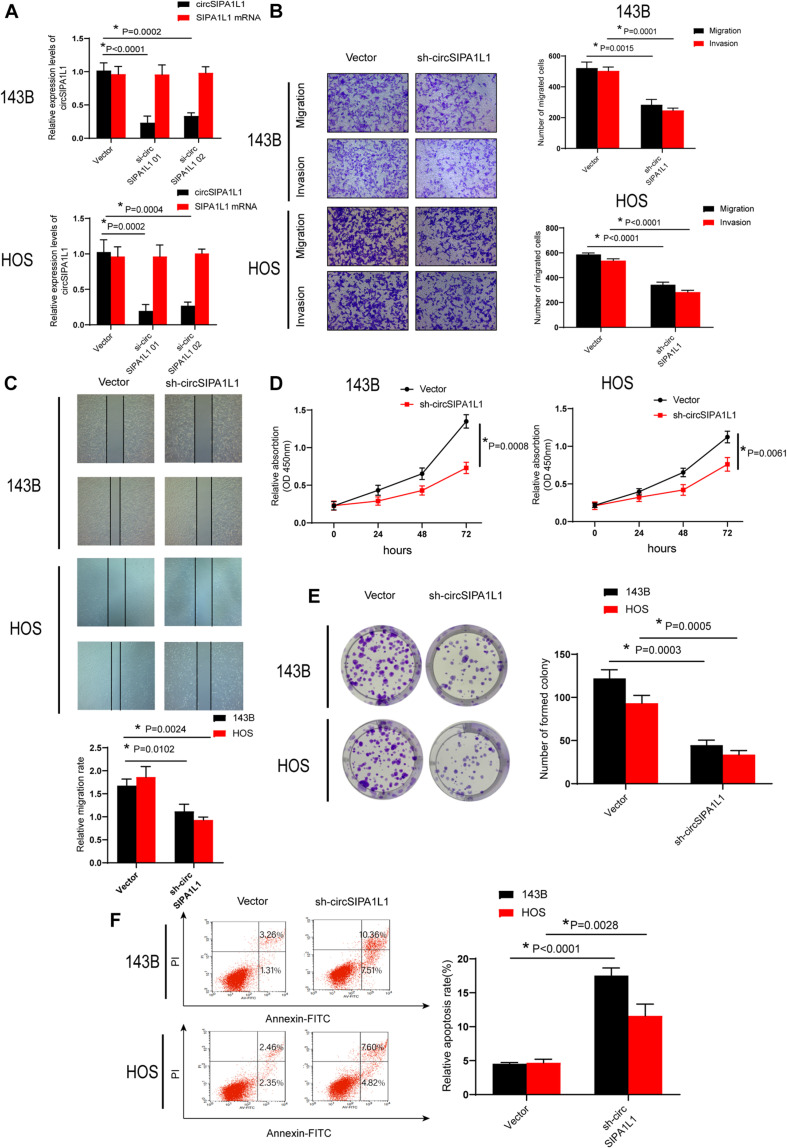
Silencing circSIPA1L1 attenuated cell proliferation, invasion, and migration in OS cells. **(A)** The expression levels of circSIPA1L1 and SIPA1L1 mRNA in 143B and HOS cells after stably transfected with N.C. (negative control) or sh-circSIPA1L1 were evaluated by qRT-PCR. **(B)** Migration-related experiments confirmed circSIPA1L1 knockdown to suppress cell migration and invasion abilities of 143B and HOS cells. **(C)** Wound-healing assay was used to determine cell migration capacity of the stable 143B and HOS cells. **(D)** shRNA-mediated circSIPA1L1 knockdown attenuated OS cell growth, as confirmed by CCK-8 experiments in 143B and HOS cells. **(E)** Colony formation assay was performed to measure the cell growth capacity of transfected 143B and HOS cells. **(F)** Both 143B and HOS cells were transfected with sh-circSIPA1L1, followed by Annexin V-FITC/PI staining. Data from 3 independent experiments are presented as mean ± SD. *P < 0.05.

To identify the potential role of circSIPA1L1 in OS cells, circSIPA1L1 shRNA, which could silence the circSIPA1L1 expression, was transfected into OS cells. The sh-circSIPA1L1 product specifically aimed the junction site of circSIPA1L1 and constructed stable circSIPA1L1-silencing OS cells. Figure 2A demonstrates that the expression of circSIPA1L1 was notably decreased by si-circSIPA1L1 01 or si-circSIPA1L1 02, while its mRNA level remained unchanged. We then selected si-circSIPA1L1 01 and constructed shRNA. Accordingly, the migration and invasion capabilities of OS cell lines decreased sharply upon transfection with sh-circSIPA1L1, as observed by wound-healing assay (conducted subsequently; Figures 2B,C). The results of CCK-8 (Cell Counting kit-8) assay indicated circSIPA1L1 knockdown to remarkably compromise cell viability (Figure 2D). Consistently, as shown in Figure 2E, circSIPA1L1 silencing impaired the proliferation capacity of OS cells. Flow cytometric assays were performed to demonstrate the impact of circSIPA1L1 silencing on the survival ability of OS cells (Figure 2F). Taken together, the findings indicated that circSIPA1L1 exerted a significant influence on the biological behavior of OS cells *in vitro*.

### circSIPA1L1 Functioned as a Molecular Sponge for miR-411-5p in OS

**FIGURE 3:**
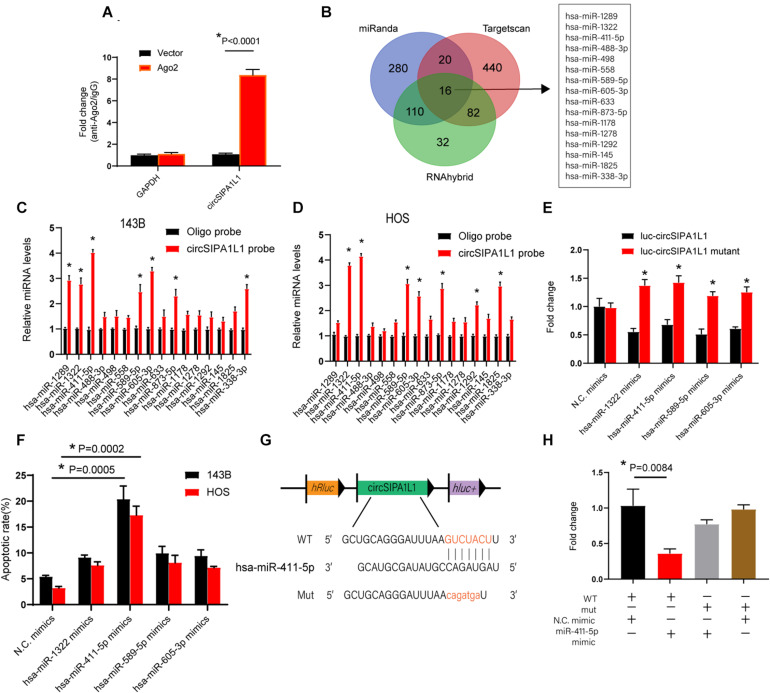
circSIPA1L1 functioned as a miR-411-5p sponge in OS progression. **(A)** The expression levels of circSIPA1L1 in circSIPA1L1 knockdown 143B cells were determined by RIP assays. **(B)** Schematic illustration presenting overlapping of the downstream miRNAs of circSIPA1L1 predicted by Targetscan, miRanda, and RNAhybrid. **(C,D)** qRT-PCR detected the relative miRNA levels of 16 miRNA candidates in the 143B and HOS lysates. **(E)** Luciferase reporter assays were employed to measure the luciferase activities of 293T cells co-transfected with mimic N.C. or miRNA mimics. **(F)** Apoptosis assay showed the apoptotic percentage of 143B and HOS cells transfected with mimic N.C. or miRNA mimics. **(G)** Schematic illustration depicting that circSIPA1L1 shares a miRNA response element with miR-411-5p. **(H)** 293T cells were co-transfected with miR-411-5p mimics (or mimic N.C.). Data from three independent experiments are presented as mean ± SD. *P < 0.05.

Previous reports suggested that circRNAs could serve as sponges for their downstream miRNAs to regulate gene expression, which could further alter cancer progression. As shown above, circSIPA1L1 was identified to predominantly locate in the cytoplasm, representing striking stability. We hypothesized that circSIPA1L1 might serve as an efficient sponge for miR-411-5p. To check the hypothesis, we subsequently performed RIP assay for AGO2 in 143B cells. Figure 3A showedendogenous circSIPA1L1 pulled down by AGO2 antibodies to be remarkably enriched in the AGO2 overexpression group when compared with the control group. These results indicated that circSIPA1L1 interacted and bound to miRNAs through AGO2 protein. To identify whether circSIPA1L1 functioned as a miRNA sponge in 143B and HOS cells, we utilized three biological databases (RNAhybrid, miRanda, and TargetScan) to predict the underlying target miRNAs of circSIPA1L1. Sixteen candidate miRNAs were picked out by overlapping the prediction diagrams (Figure 3B). Next, we designed a biotinylated circSIPA1L1 probe and conducted a pull-down assay, based on which five probable miRNAs with greatly increased fold-changes were selected in both 143B and HOS cells (Figures 3C,D). A luciferase assay was subsequently performed, and the binding of four miRNAs was verified (Figure 3E).

An apoptosis experiment was conducted to explore the specific role of miRNAs in OS cells. Figure 3F indicates that among the multiple miRNAs, overexpression of miR-411-5p caused the highest apoptotic rates in both 143B and HOS cells. Bioinformatic analysis revealed a miRNA response element that circSIPA1L1 shares with miR-411-5p (Figure 3G). Subsequently, we mutated this miRNA response element using a luciferase reporter including circSIPA1L1 in the 3′-untranslated region (3′-UTR). We discovered the luciferase activity of mut (mutant) to be remarkably higher than that of the wild-type (WT) reporter (Figure 3H), hence indicating the ability of miR-411-5p to bind to circSIPA1L1 directly. Collectively, the above results indicated miR-411-5p to be sponged by circSIPA1L1.

### Knockdown of miR-411-5p Reversed the sh-circSIPA1L1-Induced Attenuation of Malignant Behavior of OS Cells

**FIGURE 4:**
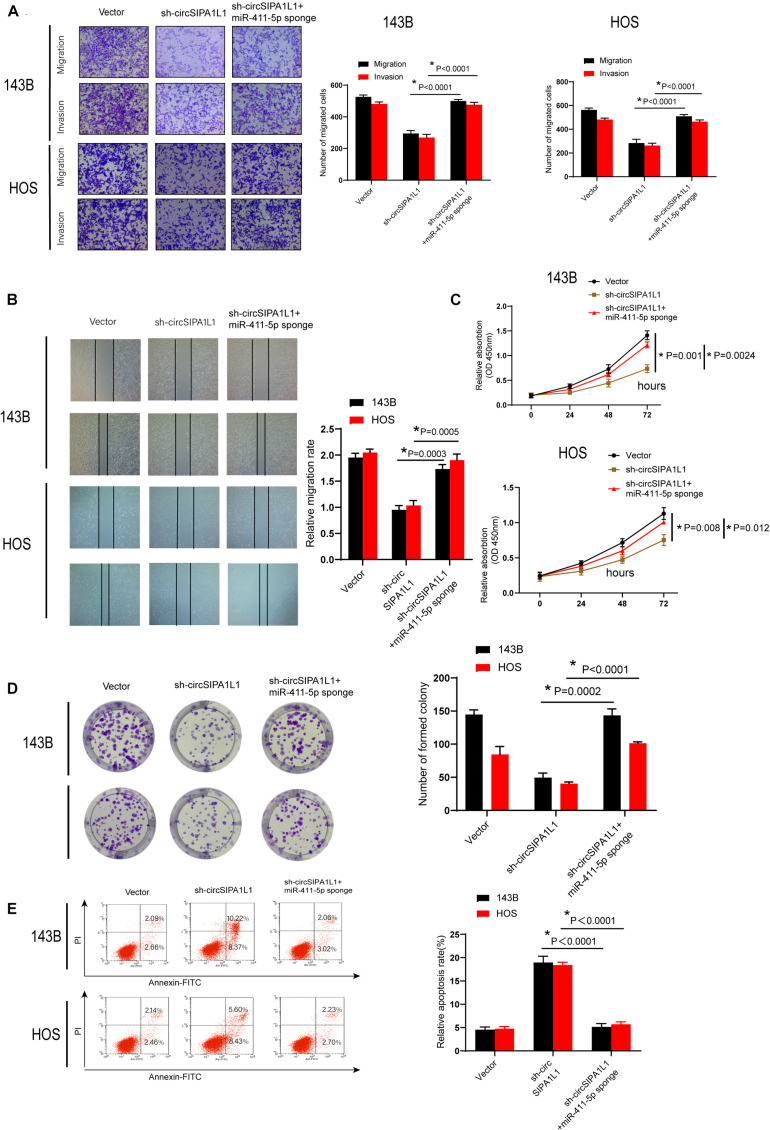
Knockdown of miR-411-5p recovered sh-circSIPA1L1-induced impairment of cell growth, invasion, and migration of OS cells. **(A)** Both 143B and HOS cells were stably transfected with N.C., sh-circSIPA1L1, or co-transfected with sh-circSIPA1L1 and miR-411-5p sponge. Migration-related assays were employed to evaluate the cell invasion and migration abilities of OS cells. **(B)** Wound-healing experiment validated the recovery of migration capacity by miR-411-5p sponge. **(C)** Downregulation of circSIPA1L1 and miR-411-5p during cell proliferation was confirmed by CCK-8 assays. **(D)** Effects of circSIPA1L1 silencing on OS cell growth were removed by miR-411-5p inhibition. **(E)** Apoptosis assay demonstrated the reversal of cell survival ability by miR-411-5p sponge. Data from 3 independent experiments are presented as mean ± SD. *P < 0.05.

We further performed a series of rescue experiments to investigate whether circSIPA1L1 could bind and interact with mir-411-5p to amplify malignant behavior in OS. sh-circSIPA1L1 and mir-411-5p sponges were co-transfected in both 143B and HOS cells. Attenuation of cell proliferation, migration, invasion, viability, and survival ability was blocked by the exogenous knockdown of miR-411-5p, as shown by the results of wound-healing, Matrigel invasion, transwell migration, CCK-8, colony formation, and apoptosis experiments (Figures 4A–E). These results together demonstrated that circSIPA1L1 mediates OS progression by sponging miR-411-5p *in vitro*.

### miR-411-5p Targeted RAB9A in OS

**FIGURE 5:**
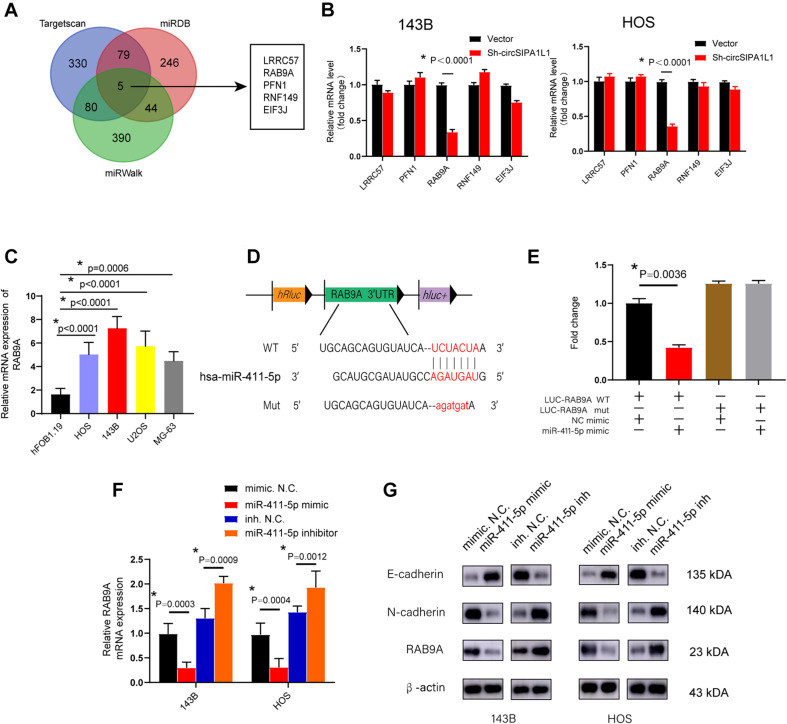
*RAB9A* serves as a target gene of miR-411-5p. **(A)** Schematic illustration exhibiting overlapping of the downstream genes of miR-411-5p, as determined by TargetScan, miRDB, and miRWalk. **(B)** qRT-PCR was utilized to evaluate the expression of five selected genes in OS cells transfected with sh-circSIPA1L1 or N.C. **(C)** qRT-PCR was conducted to assess the expression of RAB9A in OS cell lines. **(D)** Schematic illustration exhibiting the complementary sequence between miR-411-5p and RAB9A. **(E)** 293T cells were co-transfected with miR-411-5p mimics (or mimic N.C.). **(F)** The mRNA level of *RAB9A* in OS cells transfected with miR-411-5p mimic or miR-411-5p inhibitor was detected using qRT-PCR. **(G)** Western blotting was employed to evaluate the capacity of miR-411-5p to influence the expression of E-cadherin, N-cadherin, and RAB9A at the protein level. Data from three independent experiments are presented as mean ± SD. *P < 0.05.

After identifying that miR-411-5p could partly restore the impairment of malignant tumor phenotype caused by sh-circSIPA1L1. We further explore the specific mechanism of miR-411-5p in OS progression. We selected TargetScan, miRDB, and miRWalk databases to predict the possible targets of miR-411-5p. Five genes (*LRRC57*, *RAB9A*, *PFN1*, *RNF149*, and *EIF3J*) were selected by overlapping the prediction results (Figure 5A). Expression of these five genes was detected in 143B and HOS cells transfected with sh-circSIPA1L1. qRT-PCR results indicated circSIPA1L1-knockdown to notably decrease the expression of RAB9A in both 143B and HOS cells (Figure 5B). To investigate whether RAB9A was upregulated in OS cell lines, qRT-PCR was performed and the results demonstrated the high expression level of RAB9A in OS cell lines compared with hF0B 1.19 cells (Figure 5C). We further explored whether miR-411-5p could directly interact with RAB9A. The RAB9A 3′-UTR possessed sequences complementary to the miR-411-5p seed sequence (Figure 5D). Therefore, we generated 3′-UTR sensors and co-transfected 293T cells with miR-411-5p mimics. Reduced luciferase activity due to the RAB9A 3′-UTR was detected using miR-411-5p mimics. Accordingly, much higher luciferase activity was observed using mutated forms of the RAB9A 3′-UTR (Figure 5E). qRT-PCR and western blotting were performed to investigate whether RAB9A could be directly targeted by miR-411-5p. As expected, miR-411-5p overexpression in co-transfected 143B and HOS cells decreased RAB9A expression at both mRNA and protein levels, whereas miR-411-5p inhibition in the cells showed the opposite effects on RAB9A expression at both mRNA and protein levels (Figures 5F,G). Together, the results suggested *RAB9A* to be an oncogene in OS and to possibly be targeted by miR-411-5p directly.

### circSIPA1L1 Mediated OS Progression via RAB9A

**FIGURE 6:**
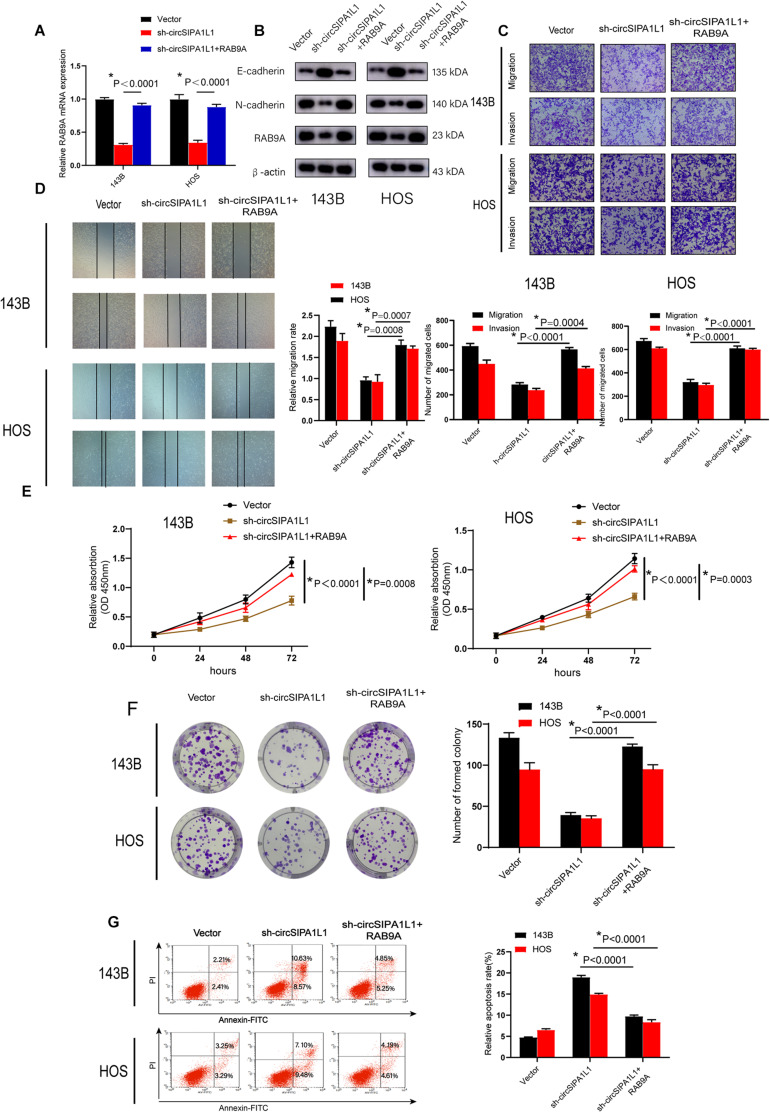
*RAB9A* functions as an oncogene in OS. **(A)** qRT-PCR demonstrated that overexpression of RAB9A could restore the decrease of *RAB9A* mRNA expression induced by sh-circSIPA1L1 in 143B and HOS cells. **(B)** Expression of N-cadherin, E-cadherin, and RAB9A at the protein level in 143B and HOS cells was determined using western blotting. Cells were transfected with vector or sh-circSIPA1L1, with or without RAB9A overexpression. **(C)** Cell migration and invasion abilities of OS cells transfected with control vector or sh-circSIPA1L1, with or without RAB9A overexpression, were determined by migration and invasion assays. **(D)** RAB9A overexpression accelerated the migration of sh-circSIPA1L1-mediated cells, as determined by a wound-healing assay. **(E)** The effects of circSIPA1L1 knockdown and RAB9A overexpression on cell vitality were determined by a CCK-8 assay. **(F)** The proliferation capacity of stable 143B and HOS cells was detected by a colony formation assay. **(G)** Apoptosis assay demonstrated the alteration of cell survival ability of 143B and HOS cells under the effects of circSIPA1L1-deficiency and RAB9A-overexpression. Data from three independent experiments are presented as mean ± SD. *P < 0.05.

To further identify whether circSIPA1L1 contributes to OS progression through RAB9A, we established an RAB9A overexpression plasmid and transfected it into circSIPA1L1-deficient OS cells. qRT-PCR data indicated RAB9A to partly reverse the decrease in RAB9A expression resulting from circSIPA1L1 silencing (Figure 6A). Western blotting confirmed this effect at the protein level. Intriguingly, change in E-cadherin and N-cadherin expressions also confirmed the crucial role of RAB9A in recovering the loss of EMT caused by circSIPA1L1-deficiency in cells (Figure 6B). To investigate whether RAB9A was able to recover the phenotypes of circSIPA1L1-knockdown OS cells, rescue experiments were performed. Cell migration and invasion of OS cells were found to be significantly restored by RAB9A (Figures 6C,D). In addition, increasing RAB9A levels abrogated the impairment of cell viability and colony-forming ability of OS cells caused by sh-circSIPA1L1 (Figures 6E,F). Moreover, apoptosis assay revealed OS cell survival ability to be ameliorated by the overexpression of RAB9A (Figure 6G). Taken together, the results indicated the regulation of OS progression by circSIPA1L1 via targeting RAB9A.

### The Role of circSIPA1L1 in Tumor Growth *in vivo*

**FIGURE 7:**
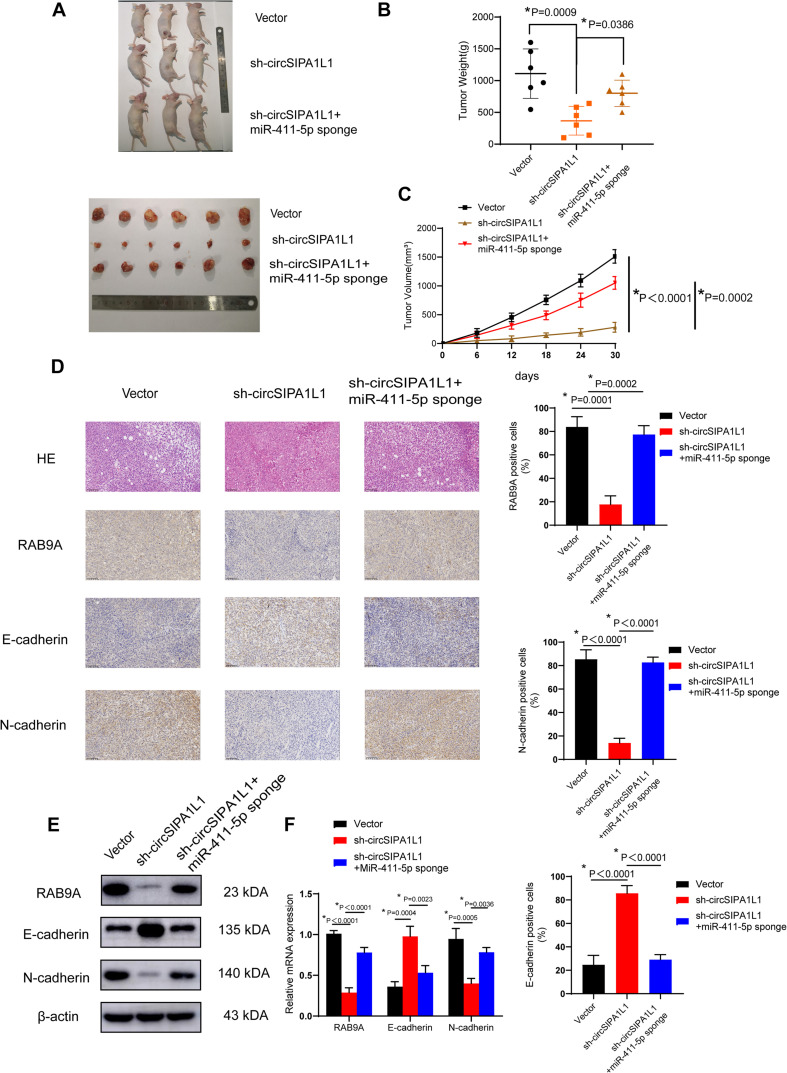
circSIPA1L1 serves as a miR-411-5p sponge to accelerate OS progression *in vivo*. **(A)** Nude mice were subcutaneously injected with 4 × 10^6^ 143B stable vector cells, or cells transfected with sh-circSIPA1L1, or cells co-transfected with sh-circSIPA1L1 and miR-411-5p sponge. Thirty days after injection, animals were dissected and photographed. **(B)** Tumor weight was determined on the day the animals were sacrificed (*n* = 6). **(C)** Tumor volumes (*v* = width × length)^2^/2 were measured and calculated from the day the mice were injected with stable 143B cells (*n* = 6). **(D)** Histological analysis by H & E staining and immunohistochemistry. Data from three independent experiments are presented as mean ± SD. *P < 0.05. **(E)** Western blotting analysis of RAB9A, E-cadherin and N-cadherin in tumor samples. **(F)** qRT-PCR identified the mRNA expression of RAB9A, E-cadherin and N-cadherin in tumor samples from different groups.

**FIGURE 8:**
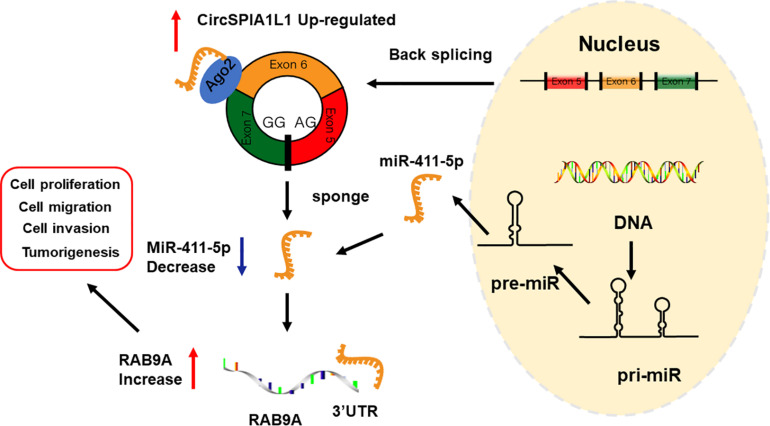
Schematic illustration of the circSIPA1L1/miR-411-5p/RAB9A axis.

A xenograft model was constructed to explore the roles of circSIPA1L1 and mir-411-5p *in vivo*. 143B cells, stably expressed with negative control or circSIPA1L1-deficient or co-expressed with sh-circSIPA1L1 and the mir-411-5p sponge were established and subcutaneously injected into nude mice. The tumors harvested from the circSIPA1L1-knockdown group were found to be distinctly inferior, in both volume and weight, to those from the control group. Intriguingly, the miR-411-5p sponge remarkably reversed the circSIPA1L1-induced antitumor effects on volume and weight (Figures 7A–C). Intriguingly, according to the results of IHC, knockdown of circSIPA1L1 remarkably impaired the mesenchymal phenotype of OS, while miR-411-5p sponge showed the opposite effect (Figure 7D). What’s more, western blotting and qRT-PCR confirmed these results at the mRNA and protein level (Figures 7E,F). To sum up, these results collectively demonstrate that circSIPA1L1 might exert a significant influence on OS progression *in vivo* (Figure 8).

## Discussion

As a newly emerging member of the non-coding RNA family, circRNAs have gained extensive attention recently, owing to the advances in bioinformatics approaches and widespread use of transcriptome sequencing (Xiong et al., 2019; Li R. et al., 2020). Accumulating research has suggested that circRNAs are involved in a variety of disease processes owing to their unique characteristics, such as high abundance, structural stability, and conservation across species (Zhang et al., 2018; Li Y. et al., 2019). Besides the abnormal activation of oncogenes and inactivation of antioncogenes, circRNAs also exert a significant effect on the genesis, proliferation, and metastasis of tumors (Arnaiz et al., 2019). Nevertheless, the detailed functions and mechanisms of these circRNAs still remain unclear. In this research, we concentrated on the specific functions and potential mechanisms of circSIPA1L1, which is enhanced in OS cell lines and tissues.

circSIPA1L1 originates from the signal induced proliferation associated 1 like 1 (*SIPA1L1*) gene (Wang et al., 2017). Previous studies had confirmed SIPA1L1 to be strongly correlated with colorectal cancer and hereditary non-polyposis (Isobe et al., 2017; Ge et al., 2020). In our current study, expression of circSIPA1L1 was confirmed to be upregulated in both OS cell lines and tissue. In addition, the stability of circSIPA1L1 was determined after treatment with RNase R. Silencing of circSIPA1L1 significantly attenuated the malignant behavior of OS cells, as determined by loss-of-function assays. Collectively, these findings suggested that circSIPA1L1 plays an important part in the pathogenesis of OS.

Circular RNAs are acknowledged to be capable of regulating gene expression through a variety of regulatory models, such as sponging microRNA, interacting with RNA-binding proteins, and contributing to protein translation. Among the multiple regulatory models, microRNA sponging is the most classical and most frequently reported. circRNAs, as ceRNAs, possess miRNA-binding sites and can serve as natural miRNA inhibitors to regulate gene expression. For instance, the CDR1/ciRS-7 circRNA harbors over 70 conserved binding sites specifically for miR-7 and is able to alter the expression of miR-7 target gene as an miR-7 sponge (Memczak et al., 2013). Similarly, has_circ_0030998 downregulates miR-558 expression as a sponge to suppress lung cancer tumorigenesis (Li X. et al., 2020). Other studies by our group had also demonstrated that circSIPA1L1 exhibits post-transcriptional regulation as miR-411-5p sponge to recover the expression of RAB9A, which is also targeted by miR-411-5p. In the current study, 16 candidate miRNAs targeted by circSIPA1L1 were selected using bioinformatic analysis. The interactions between circRNAs and miRNAs were confirmed by RIP and luciferase assays. Upon further investigation, miR-411-5p was found to exhibit the strongest binding ability with circSIPA1L1 among the various candidate miRNAs. The rescue assays performed subsequently confirmed the effects of miR-411-5p on OS both *in vivo* and *in vitro*. Besides, according to the results of bioinformatic analysis and luciferase report assay, we discovered that miR-411-5p and RAB9A share a complementary binding sequence, and RAB9A could be directly targeted by miR-411-5p to alter OS progression. We also confirmed this combination at the protein and mRNA level. In addition, our study indicated circSIPA1L1 to mediate the biological processes of OS via the miR-411-5p/RAB9A axis. RAB9A is a subtype of RAB9, which plays a significant role in the biological process of breast cancer cells; silencing of RAB9 can attenuate the malignant phenotypes of melanoma cells. As far as I know, this is the first report of *RAB9A* being identified as an oncogene in OS using loss-of-function assays and rescue experiments. In summary, our findings demonstrated that circSIPA1L1 promotes OS progression and metastasis by regulating RAB9A expression and sponging miR-411-5p.

In the current study, we identified the role of circSIPA1L1/miR-411-5p/RAB9A axis in OS progression. Detailed mechanisms underlying the regulation of OS by circSIPA1L1 and confirmation of whether circSIPA1L1 has a strong connection with the clinical stage of OS would require further investigation.

## Conclusion

Our results confirmed circSIPA1L1 expression to be remarkably upregulated in OS cells and tissues. We demonstrated the promotion of OS progression and metastasis by circSIPA1L1 via efficient sponging of miR-411-5p to regulate RAB9A expression, which in turn has been proven to be a driver gene in OS. Our study is the first to identify the role of circSIPA1L1 in OS via the miR-411-5p/RAB9A axis, and hence, might offer a potential biomarker for early diagnosis of OS.

## Data Availability Statement

The raw data supporting the results of this article will be made accessible by the authors, without undue reservation. The circRNA microarray (GSE96964) analyzed during the current study is available in the Gene Expression Omnibus (https://www.ncbi.nlm.nih.gov/geo/).

## Ethics Statement

The studies involving human participants were reviewed and approved by the Ethics Committee of the Sir Run Run Shaw Hospital. The patients/participants provided their written informed consent to participate in this study. The animal study was reviewed and approved by Ethics Committee of the Sir Run Run Shaw Hospital.

## Author Contributions

JM and SS conceived the research. YX, TY, HN, YH, and DQ performed the experiments. YX, TY, HN, and RZ analyzed the data. JM and YX wrote the manuscript. All authors contributed to the article and approved the submitted version.

## Conflict of Interest

The authors declare that the research was conducted in the absence of any commercial or financial relationships that could be construed as a potential conflict of interest.
