# Supplementary material for: Circular RNA circSIPA1L1 Contributes to Osteosarcoma Progression Through the miR-411-5p/RAB9A Signaling Pathway
Source: Front Cell Dev Biol. 2021 Apr 22;9:642605. doi: 10.3389/fcell.2021.642605 (PMC8100523; doi:10.3389/fcell.2021.642605)
Supplement: Supplementary file 1 [file Table_1.DOCX]

Table 1 The primers of short hairpin RNA used in the cell transfection

| **Name Sequence (5′→ 3′))** |
| --- |

Si circSIPA1L1 01 AGGGAGAAAGCATGGGATT

Si circSIPA1L1 02 TGCGAGCGTAGCATACTACC

Sh-circSIPA1L1

F:

R: AATTGAAAAAAGGGAGAAAGCATGGGATTCTCGAGAATCCCATGCTTTCTCCCT

miR-411-5p mimics UAGUAGACCGUAUAGCGUACG

miR-411-5p inhibitors CGUACGCUAUACGGUCUUAUA

|  |
| --- |
