# Supplementary material for: Circular RNA circSIPA1L1 Contributes to Osteosarcoma Progression Through the miR-411-5p/RAB9A Signaling Pathway
Source: Front Cell Dev Biol. 2021 Apr 22;9:642605. doi: 10.3389/fcell.2021.642605 (PMC8100523; doi:10.3389/fcell.2021.642605)
Supplement: Supplementary file 2 [file Table_2.DOCX]

Table 2 The primers used in the Quantitative real-time PCR

| **Name Sequence (5′→ 3′))** |
| --- |

hsa_circSIPA1L1_006

Forward CCAGCTTGAAACGGTCACAGA

Reverse GCGCCGCATGTAGAAATCATC

SIPA1L1

Forward CCAGCTTGAAACGGTCACAGA

Reverse GCGCCGCATGTAGAAATCATC

GAPDH

Forward TCAAGATCATCAGCAATGCC

Reverse CGATACCAAAGTTGTCATGGA

Beta Actin

Forward CATGTACGTTGCTATCCAGGC

Reverse CTCCTTAATGTCACGCACGAT

miR-411-5p

Forward CCGGAACCCCCTCCTTACTC

Reverse AATGGGATGTGTCCGAAGGA

U6

Forward CTCGCTTCGCRCAGCACA

Reverse AACGCTTCACGAATTTGCGT

RAB9A

Forward AGGGACAACGGCGACTATC

Reverse TCTGACCTATCCTCGGTAGCA

|  |
| --- |
