# Supplementary figures and images for: Circular RNA circSIPA1L1 Contributes to Osteosarcoma Progression Through the miR-411-5p/RAB9A Signaling Pathway
Source: Front Cell Dev Biol. 2021 Apr 22;9:642605. doi: 10.3389/fcell.2021.642605 (PMC8100523; doi:10.3389/fcell.2021.642605)

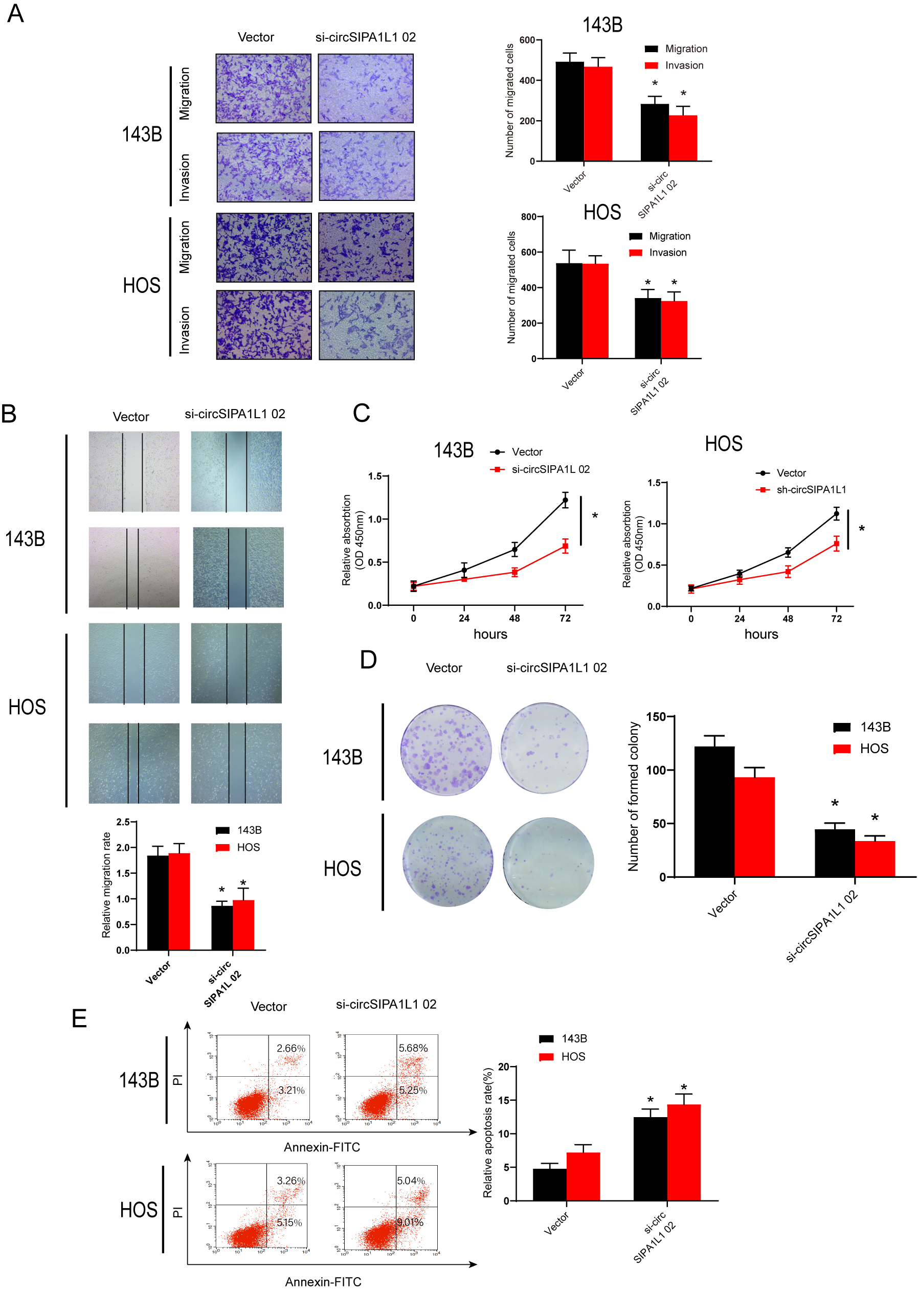

Supplement: Supplementary file 3 [file Image_1.TIF]
